# Zero-crossing patterns reveal subtle epileptiform discharges in the scalp EEG

**DOI:** 10.1038/s41598-021-83337-3

**Published:** 2021-02-18

**Authors:** Jan Pyrzowski, Jean- Eudes Le Douget, Amal Fouad, Mariusz Siemiński, Joanna Jędrzejczak, Michel Le Van Quyen

**Affiliations:** 1 Bioelectrics Lab, Institute of Brain and Spine (ICM), (UMRS 1127, CNRS UMR 7225), Pitié-Salpêtriere Hospital, 47 Boulevard de l’Hôpital, 75013 Paris, France; 2 Bioserenity, Institute of Brain and Spine (ICM), Paris, France; 3 Sorbonne University, UPMC Univ, Paris 06, 75005 Paris, France; 4 Department of Neurology, Ain-Shams University, Cairo, Egypt; 5 Department of Emergency Medicine, Medical University of Gdańsk, Gdańsk, Poland; 6 Department of Neurology and Epileptology, Medical Centre for Postgraduate Education, Warsaw, Poland; 7 Laboratoire D’Imagerie Biomédicale, (INSERM U1146UMR7371 CNRS, Sorbonne université), Campus des Cordeliers, 15 rue de l’Ecole de Médecine, 75006 Paris, France

**Keywords:** Biomarkers, Epilepsy, Diagnostic markers

## Abstract

Clinical diagnosis of epilepsy depends heavily on the detection of interictal epileptiform discharges (IEDs) from scalp electroencephalographic (EEG) signals, which by purely visual means is far from straightforward. Here, we introduce a simple signal analysis procedure based on scalp EEG zero-crossing patterns which can extract the spatiotemporal structure of scalp voltage fluctuations. We analyzed simultaneous scalp and intracranial EEG recordings from patients with pharmacoresistant temporal lobe epilepsy. Our data show that a large proportion of intracranial IEDs manifest only as subtle, low-amplitude waveforms below scalp EEG background and could, therefore, not be detected visually. We found that scalp zero-crossing patterns allow detection of these intracranial IEDs on a single-trial level with millisecond temporal precision and including some mesial temporal discharges that do not propagate to the neocortex. Applied to an independent dataset, our method discriminated accurately between patients with epilepsy and normal subjects, confirming its practical applicability.

## Introduction

Scalp electroencephalography (EEG) is performed routinely during the clinical workup of focal epilepsy^1–3^, also providing means for non-invasive localization of the epileptic focus^4^ to guide surgical treatment of pharmacoresistant patients^5^. Despite its clinical importance, the review of scalp EEG recordings requires expert readers, is time-consuming, expensive, and has not improved over many decades. Visual identification of interictal epileptiform discharges (IEDs), consisting mostly of spikes and sharp waves^3^, is hampered by limited signal-to-noise ratio (SNR) and interference from complex electrical artifacts. In selected patients, intracranial EEG recordings (iEEG) are performed using invasive depth or subdural electrodes^6,7^. This procedure radically improves SNR but increases the cost, the delay to diagnosis, and the risk of complications. Therefore, improved scalp EEG-based diagnostic methods are an area of considerable interest^8,9^.

**Figure 1:**
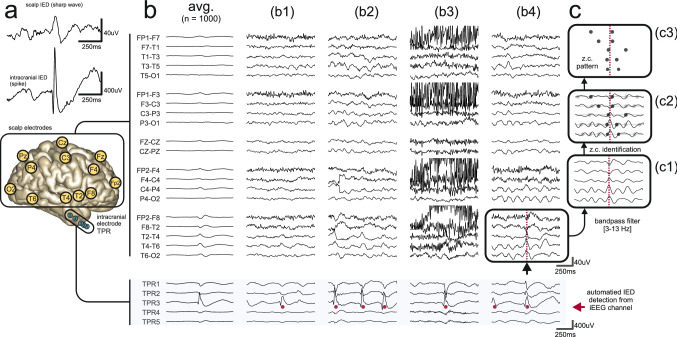
Examples of scalp EEG waveforms associated with intracranial IEDs. (**a**) Scalp IEDs are less clear than their intracranial counterparts which results in inferior SNR. (**b**) Representative examples of incomplete discharge propagation (FCD + HS subject) in simultaneous scalp EEG and iEEG recordings. For clarity, only the right lateral temporal chain of scalp electrodes is illustrated. Automatically detected intracranial IEDs are marked by red dots under the intracranial portion of the signals (light blue background). Associated scalp waveforms are below (**b1**,**b3**) or near the limit of visual identifiability (**b2**,**b4**) but all were robustly detectable as a scalp zero-crossing pattern. (**b2**) shows trial-to-trial variability. (**b3**,**b4**) show how scalp EEG artifact or background rhythms interfere with visual detection. The first column (avg.) shows a small waveform resulting from signal averaging (c.f. Fig. 3a of Koessler et al.^18^). (**c**) Transformation of a multi-channel scalp EEG signal to a zero-crossing (z.c.) pattern (**c3**) through the steps of prefiltering (**c1**) and zero-crossing detection (**c2**).

IEDs are more prominent (Fig. 1a) and up to an order of magnitude more frequent in iEEG recordings than in concurrent scalp EEG recordings. The difference results from their incomplete propagation to scalp recording sites^10–14^ which may depend on the extent of neocortical (NC) areas involved^15^. Diagnostic sensitivity is therefore limited and a single scalp EEG recording may not be sufficient for unequivocal detection of IED presence^16,17^. It has been suggested that discharges restricted to deep structures such as the mesial temporal (MT) lobe, may not be spontaneously visible in the scalp EEG at the single-trial level due to the depth and largely closed-field characteristics of their generators^18^. The ability to detect more subtle signatures of intracranial discharges from the scalp EEG would not only provide a considerable clinical advantage in the context of epilepsy but could also prove useful in the diagnostic workup of neurodegenerative disorders^19^. While multi-feature automatic signal classification has been used in this context in patients with temporal lobe epilepsy (TLE)^20^, most such techniques are plagued by poor performance and high false-positive rates^9^.

In this study, we develop and validate a new method for the detection of intracranial IEDs from scalp EEG recordings basing on a simple procedure of scalp EEG zero-crossing analysis^21,22^. This approach maintains relevant signal features^23^ while reducing low-frequency noise and effectively detrending the signal^24–27^. After developing the technique on a dataset of long-term simultaneous scalp and intracranial recordings we confirm its practical clinical applicability on an independent scalp EEG dataset. Our results suggest that scalp zero-crossing patterns extract the spatiotemporal structure of subtle scalp voltage fluctuations correlated with intracranial IEDs and provide a powerful and computationally efficient biomarker to assess scalp EEG signals with improved artifact robustness.

## Methods

### Subjects and recording procedures

Scalp EEG and iEEG recordings of two groups of subjects were studied retrospectively:

**Group A** comprised 16 TLE subjects hospitalized in the Freiburg Epilepsy Center between 2003 and 2009 (8 male and 8 female, aged 11–63 years, mean 28 years) selected from the Epilepsiae database^28^ according to the following criteria: (1) electrophysiologically confirmed presence of an epileptic focus in the temporal lobe, (2) availability of a 72 h continuous segment of dual iEEG and scalp EEG recordings with (3) full 10–20 scalp electrode coverage + T1/T2 (FT9/FT10) electrodes. Table 1 summarizes their clinical and demographic characteristics.

**Table 1:** Clinical and demographic characteristics of Group A.

Patient no	Gender	Age (yr)	Age of epilepsy onset (yr)	Seizure types	iEEG-based focus location	Etiology	Operated location	Post-operative outcome
1	M	18	11	SP/CP	Left NC/bilateral basal TL	FCD left	Left MT	Ia (24 m)
2	M	15	6	SP	Right basal TL	FCD right (MR (−))	Right TL	Ia (3 m)
3	M	42	16	SP/CP	Right NC (not iEEG confirmed)	FCD right	Right TL	Ia (3 m)
4	M	23	18	N/A	Left MT	FCD left (histology N/A)	Left TL	Ib (24 m)
5	F	22	18	SP	Left NC	FCD left	Left TL (pole)	IIa (36 m)
6	F	47	0	SP/CP/SG	Right MT/right NC	FCD + HS right	Right TL	IIIa (12 m)
7	F	32	1	CP	Left MT	FCD + HS left	Left TL	Ia (3 m)
8	M	17	1	SP/SG/CP	Left MT/left NC	FCD + HS left	Left TL	Ia (24 m)
9	M	13	0	SP/CP	Left MT/left NC	FCD + HS left	Left TL	Ia (24 m)
10	F	27	10	SP/SG	Left TL	FCD + HS left (histology N/A)	2 × left TL	Ib (3 m)
11	M	21	5	SP/CP	Right NC/right MT	FCD + HS right (MRI negative)	Right TL	Ia (12 m)
12	F	11	3	CP	Right MT	FCD + HS right (histology N/A)	Right TL	N/A
13	M	34	10	SP/CP	Right MT	FCD + HS right	Right MT	Ia (12 m)
14	F	63	30	CP/SG	Left MT/right TL	Cryptogenic, MRI (−)	Not operated	N/A
15	F	48	22	SP/CP	Bilateral TL	Cryptogenic, MRI (−)	Not operated	N/A
16	F	14	13	CP	Left TL/left NC	Cryptogenic, MRI (−) (histology N/A)	Operated (details N/A)	Ia (3 m)

*M* male, *F* female, seizure types: *SP* focal onset with preserved awareness (“simple partial”), *CP* focal onset with impaired awareness (“complex partial”), *SG* focal to bilateral tonic–clonic (“secondarily generalized”), *MT* mesial temporal, *NC* neocortical, *TL* temporal lobe, *N/A* data not available. Post-operative outcome according to Engel classification.

The signals were divided into non-overlapping epochs of 60 s length. Analysis was performed over an average of 64.8 h of recording per subject (3889 epochs, range 33.9–72 h, 2037–4320 epochs) after rejecting epochs occurring within ± 30 min of annotated seizures. 25% of these epochs, chosen at random using stratified sampling to preserve each subject’s IED frequency distribution, were reserved for the evaluation of zero-crossing pattern-based IED detection.

**Group B** comprised 46 patients (11 male and 35 female, aged 19–68 years, mean 37 years, 19 with the diagnosis of TLE and 27 nonepileptic controls) hospitalized between 2009 and 2013 in the Department of Neurology and Epileptology, Medical Center for Postgraduate Education, Warsaw, Poland. TLE subjects were selected based on the following criteria: (1) clinically certain presence of an epileptic focus in the temporal lobe (based on seizure semiology, EEG, and neuroimaging findings), (2) availability of a standard 20-min scalp EEG recording with (3) full 10–20 scalp electrode coverage. We have studied these recordings previously with different methods^27^ and the current Group B was obtained by the rejection of subjects with frontal lobe epilepsy from the dataset (see Pyrzowski et al.^27^ for further clinical and demographic information). The final 5 min (eyes closed, post-hyperventilation) of standard scalp EEG recordings with full 10–20 scalp electrode coverage were used for the analysis. No recording contained overt ictal electrographic activity. The review summaries of EEG recordings were converted to a 4-level scale (the “EEG-score”): (0) no abnormalities, (1) normal EEG variants, (2) non epilepsy-specific abnormalities (including background slowing), and (3) epilepsy-specific abnormalities (IEDs)^27^.

All data were collected during a routine clinical workup. All patients (or in the case of minors, their legal representatives) provided written statements of informed consent for research use of these data. Studies were approved by the Medical University of Gdańsk Independent Bioethics Commission for Research and the relevant bioethics commission in the Epilepsiae project (Ethik-Kommission der Albert-Ludwigs-Universität Freiburg). Data anonymization and analysis were performed in accordance with approved guidelines, and we present no information permitting the identification of subjects. All data analysis was performed in MATLAB (Mathworks Inc., 2017).

### Detection of IEDs from intracranial signals (Group A)

Signals from each intracranial channel were digitally band-passed at [0.5–70 Hz] (+ 50 Hz notch) and put into the common-average montage (with all intracranial electrodes taken into the average). Intracranial IEDs were detected automatically using a previously published algorithm^29^ with the output probability threshold fixed at 0.7. Average detection precision in a uniformly random sample of 300 detections was found to be 72% as assessed by two independent readers (A.F. and J.P., Cohen's κ = 0.63) which indicates acceptable performance^30^. It must, however, be noted that the identity of events detected with any large-scale automatic analysis must be treated with caution, especially in channels with low detection rates. From n = 1155 distinct iEEG channels analyzed (with a total of ~ 3*10^6^ detections) 26% had > 1 detection per minute and 81% with > 1 detection per hour.

An IED detected in a particular intracranial channel was considered to be “propagated” if it followed an IED of another channel by less than 200 ms (the “late propagation” window proposed by Alarcon et al.^10^). This allowed to group detections in close temporal proximity into clusters. Non-propagating MT IEDs were defined as those contained in a propagation cluster that did not extend beyond MT. This classification was naturally limited by the selective sampling of intracranial space and the inability to detect propagation in form of slow waves (as in the right column of Fig. 4b, channels BRB1-BRB2).

### Detection of zero-crossings from scalp signals (Groups A and B)

All scalp signals were digitally band-passed at 3–13 Hz and put into the longitudinal bipolar montage (Fig. 1c1). Where T1/T2 electrodes were not available (Group B), the derivations F7–T1/T1–T3 and F8–T2/T2–T4 were approximated by F7–T3 and F8–T4, respectively. Zero-crossings (crossings of the isoelectric line from electro-positive to electro-negative) were identified in each channel by linear interpolation of the signal (Fig. 1c2) to reveal zero-crossing patterns (Fig. 1c3).

### Derivation and application of zero-crossing templates

**Figure 2:**
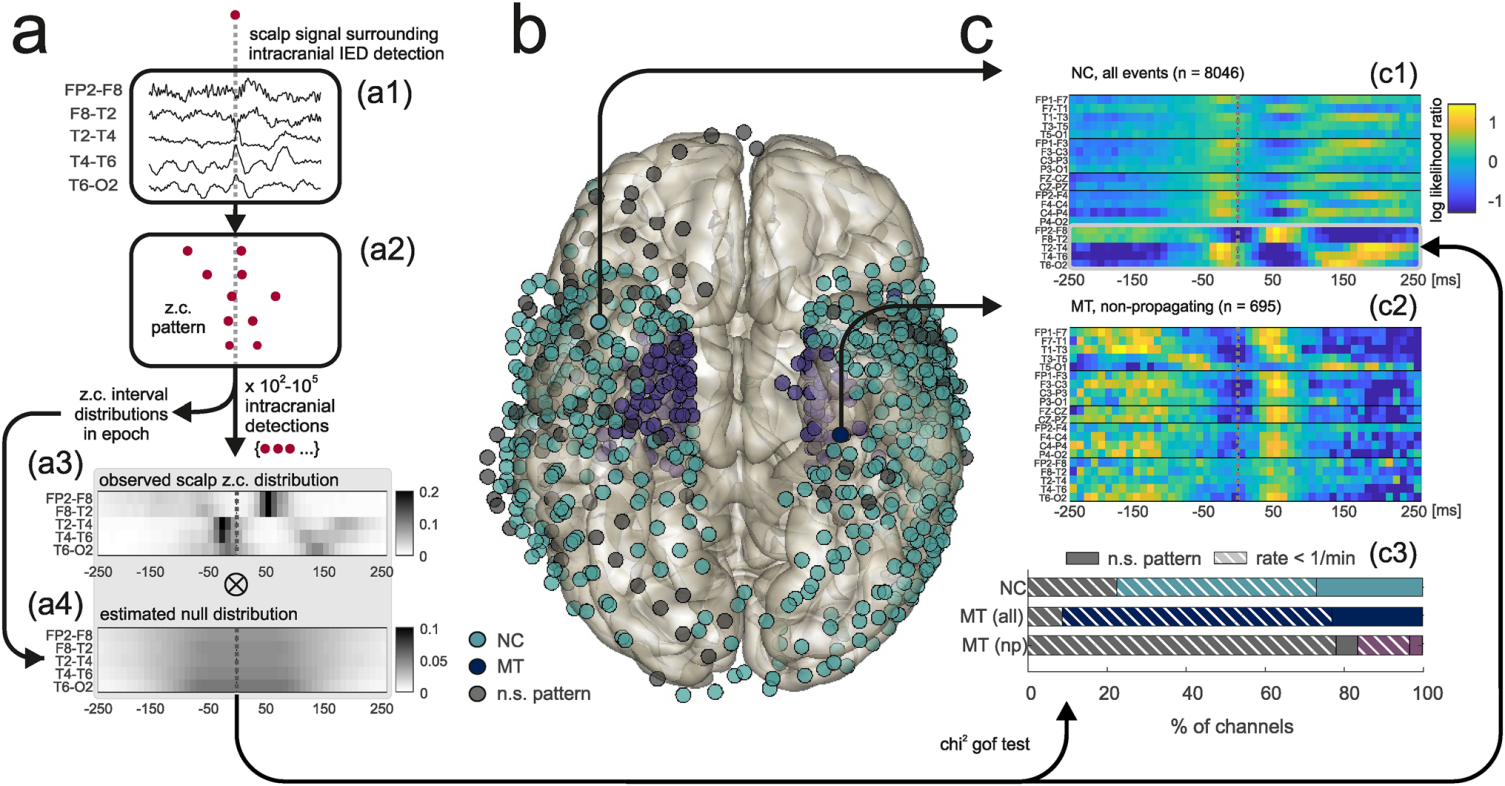
Analysis of zero-crossing patterns triggered by intracranially detected IEDs. (**a**) Derivation of a template (for clarity, only the right lateral temporal chain of scalp electrodes is illustrated). (**a1**,**a2**) Transformation of the scalp signal (**a1**) triggered by an intracranial IED detection (dotted gray line) into a zero-crossing pattern (**a2**). (**a3**) Intracranial IED detection-triggered scalp zero-crossing distribution. (**a4**) Estimated null distribution. (**b**) Intracranial IED detection-triggered zero-crossing distributions differed significantly from null distributions at both NC (light blue) and MT (dark blue) sites. Channels not associated with significant patterns are shown in gray. (**c1**,**c2**) Examples of templates derived from IEDs detected in an NC channel (**c1**) and from nonpropagating IEDs detected in a MT channel (**c2**). Color codes the pattern of log-likelihood of a scalp zero-crossing for a projected intracranial IED (dotted gray lines). (**c3**) Proportions of channels associated with significant zero-crossing patterns depending on channel location. Channels with high (> 1/min) detection rates (solid portions of bars) always gave rise to significant templates in NC/MT electrodes (light and dark blue) but not in the case of some nonpropagating MT IEDs (purple). *NC* neocortical, *MT* mesial-temporal, *np* nonpropagating.

The spatio-temporal structure of zero-crossing patterns associated with intracranially detected IEDs was summarized in form of zero-crossing templates (Fig. 2a, for details see Mathematical Appendix) representing the log-likelihood of observing a zero-crossing in a given scalp channel at a given time relative to the intracranial IED peak. One intracranial “source” channel in which IED detection was performed would give rise to exactly one template (Fig. 2c1–c2).

**Figure 3:**
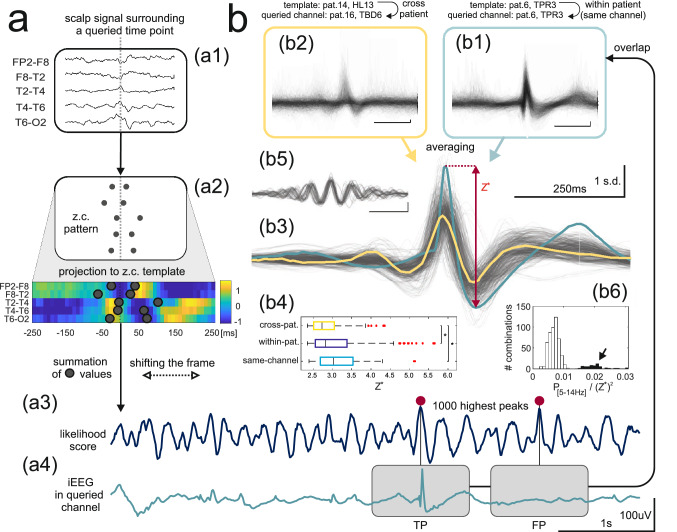
Detection of intracranial IEDs from scalp EEG. (**a**) Application of a template for intracranial IED detection basing on scalp EEG data alone. (**a1**,**a2**) Transformation of an unknown scalp signal (**a1**) centered around a queried time point (dotted gray line) into a zero-crossing pattern with subsequent ‘projection” of the pattern onto the template (**a2**) and summation of template values over zero-crossing coordinates to obtain the likelihood score. (**a3**) Shifting the frame renders the likelihood score a function of time whose peaks are then used as the times of putative intracranial IEDs. (**a4**) True positive (TP) and false positive (FP) detections are illustrated after referring back to a queried iEEG channel. (**b**) Single-trial detection and iEEG signal averaging. (**b1**,**b2**) Individual iEEG waveforms detected in two template-queried channel combinations show a high degree of overlap. (**b3**) Consistent spike-like shapes after averaging of the individual waveforms for a subset of combinations (see “Results”). Light blue and yellow correspond to averages of (**b1**,**b2**), respectively. The peak-to-peak amplitude (Z^*^) is illustrated as a measure of detection precision. (**b4**) Comparison of Z^*^ for within-patient, cross-patient, and same channel template-queried channel combinations. (**b5**,**b6**) Detection of theta and alpha rhythms (see “Results”): (**b5**) Overlap of averaged waveforms. (**b6**) Clustering of the averaged waveforms, the arrow indicates theta and alpha rhythms.

Any template could then be used in conjunction with an unknown scalp signal to assess the likelihood of the presence of an intracranial IED at a given point of time, against a null hypothesis that the momentary zero-crossings pattern is random (Fig. 3a, for details see Mathematical Appendix). The resultant running “likelihood score” was analyzed for its ability to detect IEDs in the intracranial signal (Fig. 3a4) through averaging and estimation of SNR (see Fig. 5a and the Mathematical Appendix).

## Results

### Intracranial IEDs give rise to reproducible scalp EEG zero-crossing patterns

Simultaneous recordings confirmed^10,15^ that most IEDs recorded from iEEG electrodes were not evident in the scalp EEG. However, concurrent scalp signals often revealed small waves time-locked to iEEG discharges (Fig. 1b). They were evident as subtle “notching” (Fig. 1b1) or more or less pointed waveforms in the theta-alpha frequency range (Fig. 1b2–b4) and were typically variable from trial to trial (Fig. 1b2). Without reference to an intracranial signal, however, individual scalp waveforms would likely not be interpreted as IEDs especially if obscured by artifacts (Fig. 1b3) or by superimposed background rhythms (Fig. 1b4). As observed previously^13,18^, averaging scalp EEG signals revealed a consistent low-amplitude waveform (Fig. 1b “avg.”), even for non-propagating IEDs originating from MT structures (data not shown).

Scalp signals were transformed to multi-channel time sequences of successive zero-crossings (Fig. 1c, Fig. 2a1–a2). We observed that intracranial IED-triggered scalp zero-crossing patterns captured stereotypical, amplitude-independent phase relationships that were reproducible on a single-trial level. In 79% (n = 911) iEEG channels, detection-triggered scalp zero-crossing distribution (Fig. 2a3) differed significantly from the null distribution (Fig. 2a4) indicating the stability and reproducibility of the scalp zero-crossing pattern (p < 0.05, chi-squared goodness-of-fit test with Bonferroni correction). The intracranial location of these channels is shown in Fig. 2b.

For iEEG channels that were associated with significant zero-crossing patterns the detection-triggered and null zero-crossing distributions were then combined to obtain templates (Fig. 2c, see Mathematical Appendix). Figure 2c1,c2, show representative templates derived from an NC and MT channel, respectively (Fig. 2c2 for non-propagating IEDs). Patterns of high/low likelihood were typically present in lateral-temporal scalp electrode chains ipsilaterally to the analyzed iEEG channel.

We examined the topographic consistency of this phenomenon by separating MT and NC iEEG channels. The proportion of channels associated with significant scalp zero-crossing patterns was high for both MT (91%) and NC (77%) electrodes (Fig. 2c3, dark blue and light blue) and was clearly decreased after restricting the analysis to IEDs that did not propagate from MT to NC (16%, Fig. 2c3, purple bar). Many channels with significant scalp zero-crossing patterns were associated with relatively low IED detection rates (< 1 event/min, 65% of NC channels, 75% and 80% for propagating and nonpropagating IEDs in MT channels respectively, dashed portions of colored bars in Fig. 2c3) in which cases the identity of the detected events was less certain. Only in the case of nonpropagating IEDs some MT channels with high detection rates failed to produce significant zero-crossing patterns.

### Single-trial detection of intracranial IEDs from the scalp EEG

Templates were then used to attempt detection of intracranial IEDs from the zero-crossing patterns of the scalp EEG through the calculation of a likelihood score—a readout signal with peaks at the moments of most likely intracranial IED occurrence (Fig. 3a1-a3, see Mathematical Appendix). This served not only to assess the overall performance of the method but also to confirm the IED-specificity of a given template/zero-crossing pattern. All analysis was performed on reserved recording segments that were not previously used for template derivation. Detection using a template (derived from a “source” channel) in a “queried” iEEG channel was evaluated by averaging the intracranial signal around 10^3^ highest peaks of the respective likelihood score (Fig. 3a3). All possible source-queried channel combinations were tested. In the case of cross-patient combinations (when the source channel used for template derivation belonged to a different patient than the queried channel) this can be considered as out-of-sample validation as any individual template was derived using the data from only a single subject.

Standardized iEEG waveforms extracted from the queried channel showed IED-like morphology with a significant degree of overlap for a proportion of both within- and cross-patient combinations (examples in Fig. 3b1–b2, obtained using templates in Fig. 2c1–c2, respectively). After averaging, the peak-to-peak amplitude of the resultant waveform (Z^*^) was used as a measure of detection efficiency and precision (Fig. 3b3, range indicated by the red arrow) since false or temporally imprecise detection would lead to its flattening through mutual cancellation effects.

To focus the subsequent analysis on the most reliable detection, a threshold was set at Z^*^ = 2.35 (99.95^th^ percentile of observed Z^*^ values) with n = 558 source-queried channel combinations satisfying this criterion. Out of these, n = 502 combinations showed consistent spike-like shapes of the averaged waveforms (Fig. 3b3), supporting the IED-specificity of a set of n = 101 distinct templates (which were also used throughout the subsequent sections). Within-patient (n = 299) including same-channel (n = 34) combinations corresponded to marginally higher Z^*^ values (p < 0.05, Kruskal–Wallis test with multiple comparisons, Fig. 3b4).

The remaining 10% (n = 56) combinations were found to reflect detections of rhythmic intracranial theta/alpha activity instead of IEDs (Fig. 3b5) and were easily identifiable in the distribution of the ratio of the averaged waveform’s power in the [5–14 Hz] band to the square of Z^*^ (Fig. 3b6, arrow). The associated n = 34 templates did not facilitate detection of IEDs in other channels and were rejected from further analysis.

**Figure 4:**
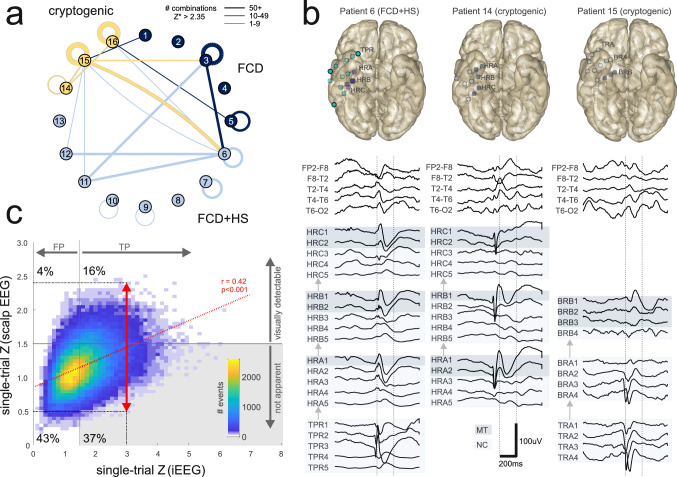
Generalization of template-based intracranial IED detection. (**a**) Cross-patient IED detection illustrated in circular network format. Link thickness represents the number of source-queried channel combinations between two given subjects. Color denotes TLE etiology, for links color is the same same as for the “source” subject. Self-links indicate within-patient detection. *FCD* focal cortical dysplasia, *HS* hippocampal sclerosis. (**b**) A fixed template detects intracranial IEDs with similar intracerebral propagation patterns (right temporal pole → right anterior hippocampus → right posterior hippocampus) in three different subjects with different TLE etiologies. The left column shows within-patient detection while the middle and right columns correspond to cross-patient detections. MT channels on a blue background, NC channels on light blue. Intracranial electrode locations are shown in upper panels. (**c**) Distribution of standardized single-trial intracranial and scalp voltage amplitudes for events detected using templates. Voltage signals were subject to filtering (0.5–70 Hz bandpass + 50 Hz notch) before analysis. Plotted scalp amplitude was the maximum over [F7–T1, T1–T3, T3–T5] or [F8–T2, T2–T4, T4–T6] for templates derived from left- or from right-hemisphere channels, respectively. Data were pooled for all supra-threshold template-queried channel combinations (see “Results”). For a fixed intracranial amplitude, scalp amplitudes were variable (red arrow) and proportional to intracranial amplitudes. Grey shaded area indicates intracranial IEDs identifiable as zero-crossing patterns but with scalp voltage amplitudes below an approximate threshold for visual detectability (Z = 1.5). *TP* true positive, *FP* false positive.

From n = 55 distinct queried channels identified in all combinations 18% (n = 10) were located in MT structures. 9 out of 16 subjects contributed to at least one cross-patient combination while another 3 contributed templates suitable only for within-patient detection (Fig. 4a). Therefore, while cross-patient combinations comprised 40% of combinations (n = 203), they were confined to only around half of the studied population (which was not found to be etiology-specific, p = 0.33, Kruskal–Wallis test).

These results support the possible generalization of zero-crossing pattern-based IED detection between different subjects with TLE. We observed that in different subjects similar scalp zero-crossing patterns tended to be associated with discharges propagating over homologous anatomical pathways. Heterogeneous intracranial electrode setups, however, make it difficult to rigorously study this association. Figure 4b shows three examples of homologous intracranial IED propagation in three patients with different TLE etiologies where all discharges were detected using the same template. While for individual events detected using templates we consistently found a correlation between single-trial simultaneous scalp EEG and iEEG amplitudes (mean r = 0.42, Fig. 4c, red dotted line) we also observed significant trial-to-trial variability (Fig. 4c, red arrow). This contrasted the relative stability of the zero-crossing patterns whose associated likelihood scores, as explained above, were always within the top 0.05% of observed values and displayed very weak correlation with simultaneous voltage amplitudes (mean r = 0.17/r = 0.12 for correlations with iEEG/scalp amplitudes, respectively).

Single-trail analysis of events detected as zero-crossing patterns, also allowed to estimate average detection specificity (~ 53%, right upper quadrant + right lower quadrant in Fig. 4c, where an approximate threshold for visual detection was set at 150% average background amplitude). The proportion of “subtle” scalp EEG discharges, detectable as zero-crossing patterns but not by visual means comprised ~ 70% of true-positives (shaded lower-right quadrant in Fig. 4c). Above-average detection performance was associated with certain template-queried channel combinations (e.g. 86% for the example in Fig. 3b1) yet with a similar proportion of “subtle” discharges (67% of true-positives resp.). These results suggest that careful application of the zero-crossing approach could, in principle, increase the yield of IED detection up to threefold compared to visual analysis alone.

### Signal-to-noise ratio assessment

**Figure 5:**
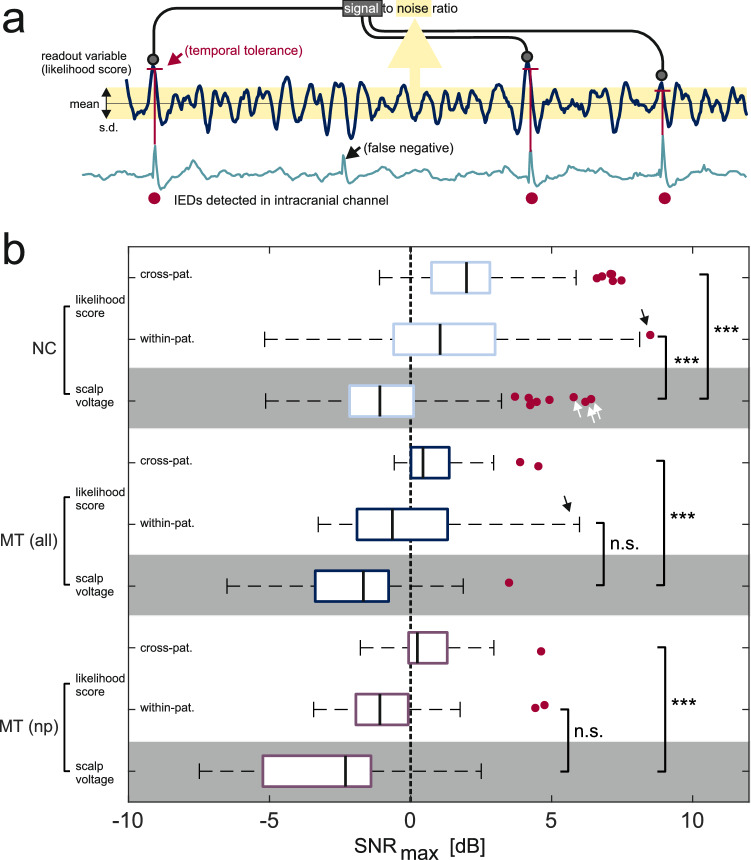
Signal-to-noise ratio assessment. (**a**) Analysis of SNR based on intracranially detected IEDs (red dots). A false negative case of automatic intracranial IED detection is illustrated. (**b**) SNR_max_ was higher for likelihood-scores (white background) than for scalp voltage-based (gray background) detection of all IED categories (NC, MT, and MT-np). Detection using cross-patient template-queried channel combinations was more effective than using within-patient combinations (in which case performance was also notably more variable, occasionally giving rise to very high SNR, black arrows). Scalp voltage also occasionally predicted intracranial NC IEDs with high SNR (white arrows) when the scalp IED expression was particularly strong. *NC* neocortical, *MT* mesial-temporal, *np* nonpropagating.

The known timing of IEDs detected from the iEEG signal in the queried channels allowed to express likelihood score-based detection efficiency through SNR values (Fig. 5a). While the signal averaging technique presented above (Figs. 3 and 4) focused on the temporal precision and specificity of detection, SNR is more a measure of detection sensitivity. We focused on channels with robustly detectable IEDs (n = 252 in NC and n = 45 in MT with > 1/min in validation epochs where n = 16 channels with false-positive detections of theta/alpha rhythms were rejected) and compared the highest SNR values obtainable using either likelihood scores or absolute scalp voltage as the readout variable (SNR_max_, see Mathematical Appendix). Likelihood scores were obtained using n = 101 templates found effective in the signal-averaging approach discussed above. The same analysis was repeated for nonpropagating MT IEDs.

Even though some of the highest scalp voltage-related SNR_max_ values were found for NC discharges (up to 6.4 dB, Fig. 5b white arrows), their median value was below zero (− 1.09 dB) indicating that IEDs arising in most iEEG channels could not be detected on a single-trial level using scalp voltage amplitude alone. This was also true for MT discharges (median − 1.67 dB) consistent with the observation that only a minority of iEEG discharges can propagate sufficiently to exceed scalp EEG background which requires the involvement of (presumably extensive) NC areas^15^. Scalp voltage-related SNR_max_ values for non-propagating-MT discharges (median − 2.3 dB) were not statistically different from SNR values reported by Koessler et al.^18^ (p = 0.29, Wilcoxon test).

In contrast, SNR_max_ values related to likelihood-scores were found to be significantly higher than their scalp voltage-related counterparts (Fig. 5b, p < 0.001, Kruskal–Wallis test with multiple comparisons). A positive median likelihood-score-related SNR_max_ value was also the case for non-propagating MT IEDs (0.24 dB), suggesting again that such discharges may be possible to detect as zero-crossing patterns at a single-trial level. Interestingly, assessment with cross-patient templates proved highly effective and was only in rare cases outperformed by within-patient templates (black arrows) whose performance was much more variable. This finding points again to the possibility of generalization of IED-related zero-crossing patterns between patients but also reflects the larger repertoire of cross-patient templates (for 13% channels a within-patient template was not available).

### External validation: epilepsy vs control classification from standard scalp EEG recordings

We next used an independent dataset of standard scalp EEG recordings to validate and test the robustness of the zero-crossing method. As in Pyrzowski et al.^27^ we addressed the power of discrimination between subjects with TLE (n = 19) and controls without epilepsy (n = 27). Since here intracranial data were not available for reference, we assumed that intracranial discharges are expected to occur in the majority of TLE subjects (even if not always apparent in the scalp EEG). Our approach followed from the previous observations: an appropriately chosen template, when applied to the scalp EEG of a TLE subject, renders prominent likelihood-score peaks associated with (and sometimes time-locked to) intracranial IEDs. Such peaks should, however, not be present if the analyzed recording came from a control subject for whom intracranial IED activity is generally not expected. Different templates were expected to perform variably in different subjects depending on how well IEDs in a given patient would fit the particular zero-crossing pattern.

**Figure 6:**
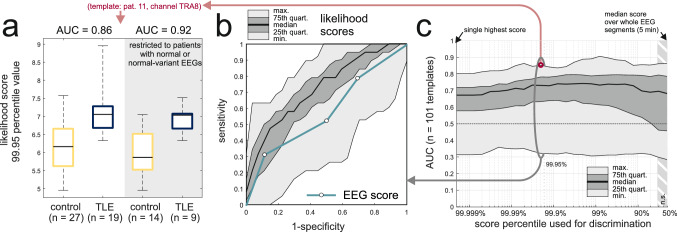
External validation: application of templates to classify TLE subjects vs controls. (**a**) Example of a template for which the 99.95th percentile values of likelihood scores discriminate between patients with TLE and control subjects also in the case when the analysis was restricted to those with normal and normal-variant scalp EEGs (grey background). (**b**) Range of AUC curves for all studied templates and likelihood score 99.95th percentile-based discrimination shown together with a ROC curve derived from clinical review of scalp EEGs (AUC = 0.58). At high-sensitivities template-based classification outperforms visual scalp EEG analysis. Both approaches perform comparably at high specificities. (**c**) Dependence of AUC values on the fixed percentile threshold. Performance of the studied set of templates was stable at AUC ~ 0.7 and significantly higher than AUC = 0.5 throughout most of the range.

Likelihood-scores were calculated for 5 min EEG segments obtained post-hyperventilation with eyes closed. Again we used the set of n = 101 templates found to be effective in the signal-averaging approach but this time the likelihood-score definition was adapted to the context of variable focus laterality (see “Methods”). The presence of peaks in each likelihood-score time series was assessed through a fixed percentile value of its amplitude distribution. The ability to discriminate between TLE and control subjects using this value (example in Fig. 6a) was assessed through receiver-operating-characteristic (ROC) curves (Fig. 6b,c).

Figure 6b shows ROC curves corresponding to the threshold set at the 99, 95th percentile (median area-under-curve, AUC = 0.72, range 0.25–0.93) along with a ROC curve based on the visual EEG assessment score (AUC = 0.59). The zero-crossing approach performed better than visual scalp EEG analysis in the high-sensitivity domain (possibly due to increased IED detection yield) and comparably in the high-specificity domain. Notably, the performance of likelihood score-based classification was stable with respect to the variation of the fixed threshold parameter (Fig. 6c). For the assessed set of templates, AUC was found to be significantly higher than 0.5 for all threshold values above the 80th percentile up to the extreme case of considering only the single highest peak of the likelihood score (p < 0.001, sign test with Bonferroni correction). Similar results were obtained in a subset of subjects with EEG recordings visually evaluated as normal or normal-variant (example in Fig. 6a, right panel). This indicates that the zero-crossing method may capture subtle TLE-specific inter-hemispheric EEG asymmetries.

## Discussion

Basing on the study of relations between concurrent iEEG and scalp EEG signals we propose the use of the zero-crossing pattern representation of scalp EEG to detect scalp signatures of intracranial IEDs. The proposed computational method has the potential to increase the clinical value of scalp EEG in epilepsy diagnosis and was externally validated on an independent dataset of standard scalp EEG recordings. Our main findings are that: (1) Zero-crossing patterns are a reliable single-trial scalp EEG biomarker of intracranial IEDs including some apparently non-propagating MT IEDs (median likelihood score-related SNR > 0 dB). (2) Most scalp EEG waveforms associated with reproducible zero-crossing patterns are amplitude-wise below background (median scalp voltage related SNR < 0 dB). (3) Detection based on zero-crossing patterns can achieve high temporal precision, be generalized between patients as well as discriminate successfully between EEG recordings from patients with epilepsy and control subjects without the need to access iEEG even in the absence of overt scalp IEDs.

Our findings suggest that subtle low-amplitude discharges associated with a reproducible zero-crossing pattern may represent a novel pathological scalp EEG abnormality. While they certainly do not meet the conventional criteria of spikes and sharp waves used for visual EEG assessment^31^, their close temporal relationship to intracranial IEDs suggests they should be explored as a putatively equivalent epilepsy biomarker. In this respect, our approach is complementary to previous methods aimed at automatized detection of visually identifiable scalp IEDs^8^. In contrast to the use of sophisticated machine-learning approaches^20^ zero-crossing patterns are well-defined events with topographic distributions over the scalp. Zero-crossing analysis has previously been used in various biological contexts including epileptology^24–27^, sleep studies^32–35^, cognitive science, and neuropsychiatry^36–39^. Zero-crossing representations may help avoid certain artifacts of scalp EEG signals^27^. Clearly, transforming scalp EEG signals to zero-crossing sequences discards amplitude-related information, but has been also proven to preserve important information characterizing the underlying dynamical system^23^. Due to effective signal compression, this detection method can be implemented with sufficiently fast execution time to operate in real-time in a continuous-monitoring environment.

Contributions of MT sources to scalp EEG have been debated since the advent of invasive EEG recordings^6,13,18^. Our results challenge the view that single non-propagating intracranial discharges derived from MT sources cannot at all be detected in the scalp EEG^18^. While we confirmed this observation for voltage amplitudes our study suggests that scalp zero-crossing patterns associated with some of these events can be reliably identified at a single-trial level. Our analysis is, however, importantly limited by incomplete coverage of intracranial space with depth electrodes, allowing some propagation pathways to be missed altogether. As this limitation applies to all studies using iEEG data, it may be questionable to what extent can we be certain that MT IEDs are indeed nonpropagating.

A related finding is that intracranial discharges of similar amplitude yield scalp events of significantly varying amplitude, many of which are too small for visual detection. Visually identifiable scalp EEG IEDs may then be viewed as only “a tip of an iceberg” of IEDs generated in neocortical and deeper brain regions. This variability points to a crucial role of active neuronal propagation in the generation of visually identifiable IEDs while volume conduction may contribute to a high temporal fidelity observed for some propagated discharges. Both mechanisms likely play a role in the complex blend of observed scalp EEG activity.

We speculate that reproducible zero-crossing patterns may reflect homologous propagation pathways in different TLE subjects. Abnormal propagation pathways may be a significant component of epileptogenic networks—a core pathology of an epileptic brain^40^. Indeed, detection based on zero-crossing patterns appears to generalize between subjects and not to depend on etiology whereas “blind” application of the method to independent EEG data permitted reliable discrimination between patients with TLE and control subjects.

Other limitations of our approach include the inability to detect intracranial IED propagation in forms other than spikes (e.g. slow waves) as well as the general limitations imposed by unsupervised/semi-supervised automatic processing of large volumes of data. In particular, the proposed method needs to be further validated through systematic comparison to standard visual EEG assessment in both scalp and iEEG signals. To confirm its localizing value and specificity, the method needs also to be assessed in epileptic syndromes other than TLE (frontal lobe epilepsy, which was underrepresented in our dataset, would be of prime interest) as well as in patients with non-epileptogenic intracranial lesions. Further study is also needed to assess whether the complex information contained in individual templates permits the reconstruction of deep sources and propagation pathways. This ability to distinguish deep and superficial sources would be an important step towards automated, noninvasive localization of an epileptic focus.

In summary, the proposed method seems appropriate for retrospective or online clinical EEG monitoring. Further rigorous tests, in a clinical setting, are needed to assess its performance and the actual computational requirements.

## Supplementary Information

Supplementary Information

## Data Availability

The dual EEG dataset analyzed during the current study (group A) is available in the Epilepsiae repository, http://www.epilepsiae.eu/. The validation dataset (Group B) is not publicly available but can become available from the corresponding author on reasonable request with the permission of JJ.
