# Supplementary material for: Zero-crossing patterns reveal subtle epileptiform discharges in the scalp EEG
Source: Sci Rep. 2021 Feb 18;11:4128. doi: 10.1038/s41598-021-83337-3 (PMC7892826; doi:10.1038/s41598-021-83337-3)
Supplement: Supplementary file 1 — Supplementary Information [file 41598_2021_83337_MOESM1_ESM.pdf]

**Zero-crossing patterns reveal subtle epileptiform discharges in the scalp EEG.**

Jan Pyrzowski, Jean-Eudes Le Douget, Amal Fouad, Mariusz Sieminski, Joanna Jędrzejczak, Michel Le Van Quyen.

**Supplementary Information**

## Mathematical Appendix

### Derivation of templates (Group A).

A template $L_k(t-t_0)$ is defined as the log-likelihood ratio of observing a zero-crossing in channel $k$ at time $t$, if an intracranial IED was to occur at $t_0$ against a null hypothesis that the observed zero-crossings were generated by a renewal process.

For every IED detected (at time $t_{IED}$) in an intracranial "source" channel the times from the previous and to the next zero-crossing ($\Delta t^{prev} = t_{IED} - t^{prev}$ and $\Delta t^{next} = t^{next} - t_{IED}$) were calculated independently for every scalp channel (Fig. 2 a1-a2). These were then histogrammed in 10ms bins to estimate the observed waiting time distributions $W_{jk}^{prev/next}$ in a given scalp channel $k$ and epoch $j$ (Fig. 2 a3).

Using the empirical distribution of time intervals between two subsequent zero-crossings $P_{jk}^{interv}$ as the "holding times" of the assumed renewal process (see also Pyrzowski et al.[27] for other biomarkers derived from this distribution) a null waiting time distribution was also estimated for each channel and epoch (Fig. 2 a4):

(1) $$W_{jk}^{null}(t) = \int_{i=t}^{\infty} P_{jk}^{interv}(i)$$

which follows from the fact that the probability of a randomly chosen time point being contained in an interval of length $i$ is $iP_{jk}^{interv}(i)$. The template (Fig. 2 c1-c2) was defined as:

(2) $$L_k(t-t_0) = \log\left(\left\langle W_k^{next}(t-t_0)\right\rangle\right) - \log\left(\left\langle W_k^{null}(t-t_0)\right\rangle\right) \text{ for } t > t_0$$

$$L_k(t-t_0) = \log\left(\left\langle W_k^{prev}(t_0-t)\right\rangle\right) - \log\left(\left\langle W_k^{null}(t_0-t)\right\rangle\right) \text{ for } t < t_0$$

where $W_k^{prev/next/null}$ are observed and null waiting time distributions in channel $k$ weighted-averaged over all epochs using the total number of IEDs detected in each epoch as the weighting factor.

The significance of the underlying IED-triggered zero crossing pattern was assessed by chi-squared goodness-of-fit testing between averaged empirical and null waiting time distributions. Prev/next histograms were tested channel-wise against respective null distributions and the obtained p-values were merged using Stouffer's Z-score method[41].

**Detection of intracranial IEDs from scalp signals (Group A)**

For an unknown scalp EEG signal, transformed into a zero-crossing pattern (Fig. 3 a1), a template $L$ can be used to calculate the likelihood score (the log-likelihood ratio statistic testing for the presence of an intracranial IED at $t_0$ against the null hypothesis as defined above) as follows:

(3) $$S(t_0)=\sum_k L_k\left(t_k^{prev}(t_0)-t_0\right)+L_k\left(t_k^{next}(t_0)-t_0\right)$$

where $t_k^{prev}(t_0)$ and $t_k^{next}(t_0)$ denote first zero-crossings found in channel $k$ before and after $t_0$ respectively and the sum runs over all scalp channels (Fig. 3 a2). Incrementing $t_0$ in steps allows to track the evolution of the likelihood score in time (Fig. 3 a3). Note that this procedure combines information from all scalp EEG channels to single readout value. Analysis of intracranial IED detection performance was performed on 25% reserved epochs of each patient, which were not used for template derivation (see Results for details).

The effect of varying epileptic focus and IED laterality in the TLE subgroup of Group B was accounted for through defining a composite likelihood score as:

(4) $$S_{comp}(t)=max\left(S(t),S_{flip}(t)\right)$$

where $S(t)$ and $S_{flip}(t_0)$ were obtained according to eqn. (3) using an original and modified ("flipped") version template where each scalp channel was substituted by its contralateral counterpart (i.e. Fp1→Fp2, Fp2→Fp1 … Fz→Fz, Cz → Cz, Pz → Pz). The composite likelihood score tests for the occurrence of a putative intracranial IED on either the left or right hemisphere against the same null hypothesis as before.

**Signal-to-noise ratio definitions**

The maximum SNR for a queried intracranial channel (see Results and Fig. 5a) obtainable using templates was defined as:

(5) $$SNR_{max}^{likelihood\,score} = 20\log_{10} \max_{L} \left( \frac{\langle S_L \rangle_{t \in t_{IED}} - \mu_{S_L}}{\sigma_{S_L}} \right)$$

where $\langle S_L \rangle_{t \in t_{IED}}$ denotes an average of peak likelihood scores found within ±25ms of intracranial IEDs detected in the "queried" channel, $\mu_{S_L}$ and $\sigma_{S_L}$ is the mean and standard deviation of the likelihood score $S_L$ and the maximum runs over templates $L$ associated with above-threshold performance in the signal-averaging approach (n = 101). The maximum in (5) was taken either over templates derived from all other patients (cross-patient analysis) or, if available, over templates derived from the studied patient (within patient analysis).

For comparison with visual IED detection maximum SNR was also defined for scalp voltage:

(6) $$SNR_{max}^{scalp\,voltage} = 20\log_{10} \max_{k} \left( \frac{\langle |V_k - \mu_{V_k}| \rangle_{t \in t_{IED}}}{\sigma_{V_k}} \right)$$

where the average runs, as before, over peak absolute scalp voltage values within ±25ms of intracranial IED detections, $\mu_V$ and $\sigma_V$ are the mean and standard deviation of the voltage $V_k$. and the maximum runs over all scalp channels $k$ (n = 20).

The ±25ms temporal tolerance was introduced to account for the possible delay between the peak of the intracranial IED and the peak of the readout variables (approximately the "sync" window defined by Alarcon et al.[10]). All voltage recordings were downsampled to 100Hz before analysis to match the 10ms time step used in the calculation of likelihood scores. Taking the absolute value of the voltage in eqn. (6) accounts for the choice of bipolar montage where scalp IEDs may appear as either positive or negative voltage deflections, whereas strictly positive peaks were expected in the case of likelihood scores. Since this prevented the numerators from being squared, a factor of 2 was introduced to allow for direct comparison with the results of Koessler et al.[18].
